# On whose shoulders we stand – the pioneering entomological discoveries of Károly Sajó *

**DOI:** 10.3897/zookeys.157.2044

**Published:** 2011-12-21

**Authors:** Károly Vig

**Affiliations:** 1 Savaria Museum, H–9700 Szombathely, Kisfaludy Sándor u. 9., Hungary

**Keywords:** Károly Sajó, history of entomology, diapause, Chrysomelidae, Hungary, Germany

## Abstract

The excellence of Károly Sajó as a researcher into Hungary’s natural history has been undeservedly neglected. Yet he did lasting work, especially in entomology, and a number of his discoveries and initiatives were before their time.

Born in 1851 in Győr, he received his secondary education there and went to Pest University. He taught in a grammar school in 1877–88 before spending seven years as an entomologist at the National Phylloxera Experimental Station, later the Royal Hungarian State Entomological Station. Pensioned off at his own request in 1895, he moved to Őrszentmiklós, where he continued making entomological observations on his own farm and wrote the bulk of his published materials: almost 500 longer or shorter notes, articles and books, mainly on entomological subjects.

Sajó was among the first in the world to publish in 1896 a study of how the weather affects living organisms, entitled *Living Barometers*. His *Sleep in Insects*, which appeared in the same year, described his discovery, from 1895 observations of the red turnip beetle, *Entomoscelis adonidis* (Pallas, 1771), of aestivation in insects – in present-day terms diapause.

It was a great loss to universal entomology when Sajó ceased publishing about 25 years before his death. His unpublished notes, with his library and correspondence, were destroyed in the World War II. His surviving insect collection is now kept in the Hungarian Natural History Museum, Budapest.

## Dedication

Dedicated to Géza Balás (1914–1987) for his seminal work on Károly Sajó’s life and achievements.

## A brief account of Károly Sajó’s life

**Figure 1.**
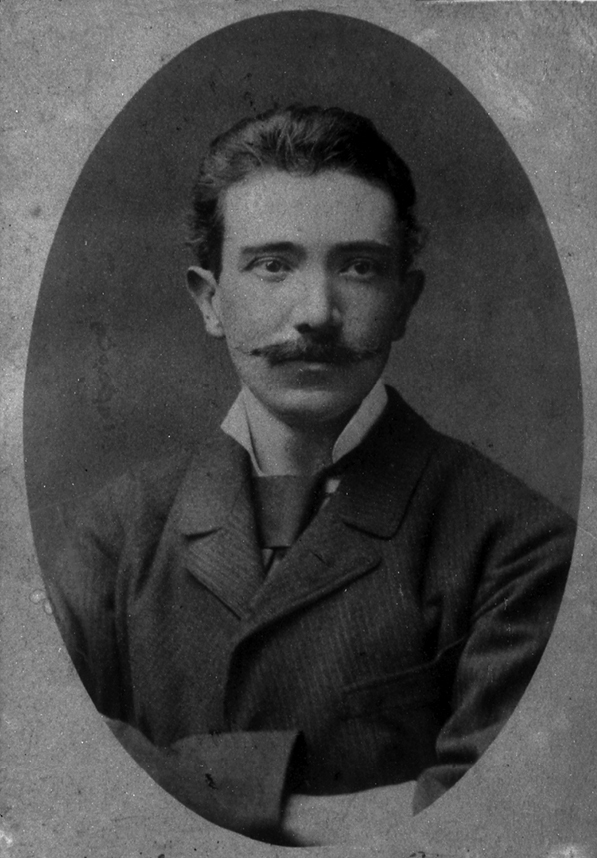
Károly Sajó (1851–1939).

The main events in the life of Károly Sajó (Figure 1) are known from a laconic biography that appeared after his death (Szent-Iványi 1941). The writings and objects he left behind were destroyed in the World War II. The same fate befell many document collections (for instance those of the Hungarian Entomological Society) in which details of his life might otherwise have been found. However, his articles and books have survived, as true reflections of the fruitful life he led.

Károly Sajó (originally Schemiz) was born in Győr on 20 June 1851. His father, Károly Schemiz had been born in Sasvár-Morvaőr (Nyitra County, now Šaštín-Stráže in Slovakia, Schoßberg-Strascha in German). Károly Schemiz the elder graduated in medicine in Vienna in 1835 and began to practise as a physician in Győr, where he died aged 53 on 4 February 1865. Contemporary comments delineated a man of noble thoughts, humanity, and many parts (Kramoliny 1865).

The son was educated at Győr Gymnasium and the Pest University, where he obtained a teaching degree in natural science, and then withdrew for three years to educate himself, gain specialist knowledge, and learn languages. He then taught at Royal Catholic High Gymnasium in Ungvár (now Uzhhorod, Ukraine) (Blanár 1913). There are plenty of publications from that period to show that he was already engaged in scientific work. In 1888, he was invited to join Géza Horváth at the National Phylloxera Experimental Station. There and in its successor institution, the Royal Hungarian State Entomological Station, he spent seven years as an entomologist (Howard 1930). However, he was retired at his own request in 1895 due to deafness caused by scarlet fever, and retired to his wife’s family estates at Kisszentmiklós (later Őrszentmiklós, now quarter of Őrbottyán), where he spent the rest of his life on scientific studies and observations. His last scientific publication appeared in 1914.

Sajó belonged to numerous learned societies, including the Hungarian Entomological Society, the Royal Hungarian Natural History Society and the Association of Economic Entomologists, as well as the Kaiserlich-königliche zoologisch-botanische Gesellschaft in Vienna and the Verein für schlesische Insektenkunde in Breslau. The yearbooks of the last show that his latest works were regularly reported at general meetings.

The wife he chose at the age of 22 was Ilona Kvassay, to whom he had family ties. They had three sons. After his first wife’s death, he was remarried to his sister-in-law, Júlia Kvassay. Both wives were sisters to the celebrated hydrological engineer Jenő Kvassay. He outlived all three of his sons. One of them, Elemér Sajó, became a talented hydrological engineer, who superintended the regulation of the Soroksár branch of the Danube and installation there of the Kvassay Lock to his own design (Filotás 2005). Elemér’s three sons and their descendants bore the surname Kvassay-Sajó.

Károly Sajó died at Őrszentmiklós on 9 February 1939. His death went largely unnoticed, although this quiet, scholarly man had gained Hungary more international renown than most of his scientist compatriots. His reputation abroad is exemplified by the fact that his likeness appeared among those of the best-known scientific writers in a feature in a German illustrated paper in the early years of the century. He (along with Raoul Francé, who always declared he was Hungarian and whom fate drove to Hungary to die) was pictured larger, at the centre of the page. How sad, indeed tragic it is that Károly Sajó, the veteran leader of German life science, should have been silenced for his style, so bold and progressive in spirit, and most of his writings neglected (Szilády 1941, Balás and Sáringer 1982, Bognár 2001). However, his years at Őrszentmiklós are still remembered in the place-name Sajó tanya (now quarter of Őrbottyán, Pest county, Hungary; http://www.historicgarden.net).

## Károly Sajó’s entomological writings: general works

Though Károly Sajó’s father had an excellent knowledge of German, Sajó knew not a word of it until he was seven. He taught himself Western languages. A remarkable knowledge of French and English is apparent in his letters, and his books and articles were enhanced by use of the best literary German. One of the main sources of his success and popularity was the way he, amidst writers debased by journalese and scientific jargon, would use the language of Goethe and Schiller (Szilády 1941).

The bulk in Sajó’s scientific writings appeared after his retirement. In the last two years before he retired, he wrote 15 articles totalling 46 pages; in the first two years after, he wrote 64 pieces totalling 282 pages. His active writing period stretched from 1872 to 1914, during which he wrote almost 500 longer or shorter contributions, brochures, reviews and books on subjects to do with entomology, general biology, agriculture, horticulture, and plant and nature protection (Balás and Sáringer 1982).

**Figure 2.**
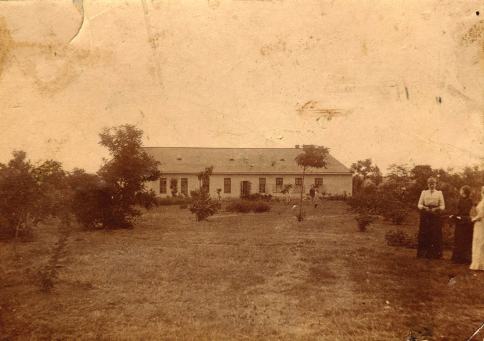
The house in the Nyáras district of Őrszentmiklós in 1891. This is where Sajó set up his laboratory.

Researchers writing about insects at the turn of the 19th and 20th centuries still practised descriptive and systematic entomology at once. Sajó dealt primarily with applied entomology. He and Géza Horváth were among the first to study insects (whether pests or not) in the context of their environment and relations with other organisms and the first to encourage their readers to do likewise. He thought it was most important to note the smallest piece of biological data, for instance the ratio of males to females in the material collected, or the dates of first and last appearance of the species. His was probably the first study of insect phenology in detail. He applied these observations on his own farm and garden (Figure 2), and his writings and advices were of much benefit to farmers at home and abroad. Naturally, the name of Károly Sajó appears alongside those of Géza Horváth and József Jablonowski as members of the Association of Economic Entomologists established in 1889. A succession of his articles about applied entomology appeared in the Hungarian and German press, as the forerunners of the new trend. It was a strange coincidence that he should have been in 1914 that he stopped publishing after three decades, just as a new scientific endeavour appeared in Europe in April that year, the *Zeitschrift für Angewandte Entomologie*. (At the same time, the seventh volume of the *Journal of Economic Entomology* was appearing in the United States.)

**Figure 3.**
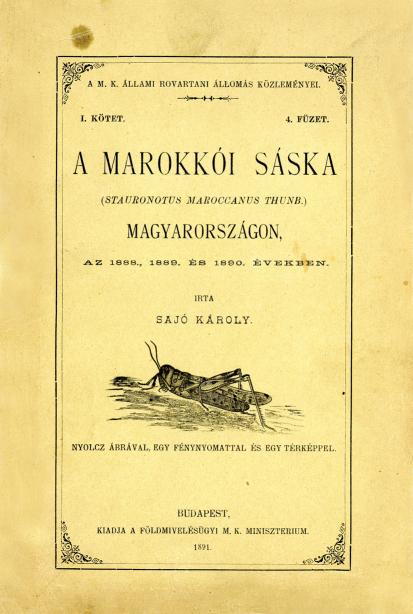
Front cover of Sajó’s book on the first outbreak of Moroccan locust, *Dociostaurus maroccanus* (Thunberg, 1815)in the Carpathian Basin, in 1888–90.

His works on horticultural entomology continue to be important sources that provide a sound basis for further research. He was the first to give accurate information on many horticultural and agricultural pests, including the cherry fruit fly, and several pests of asparagus and roses (Sajó 1895b, 1896a, 1901, 1902a, 1902b, 1902c, 1902d, 1902e, 1902f, 1903, 1904). Sajó’s years at the Royal Hungarian State Entomological Station and its predecessor coincided with the first outbreak of Moroccan locust, *Dociostaurus maroccanus* (Thunberg, 1815)in the Carpathian Basin, in 1888–90, and his works of that period provide the best account of its discovery and the practical measures taken to control it (Sajó 1889, 1890a, 1891a) (Figure 3). His articles are particularly valuable even today for an emphasis on ecological and biocoenological aspects that was well ahead of its time.

**Figure 4.**
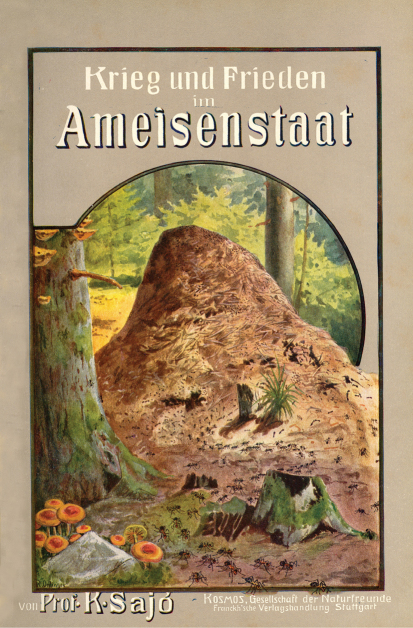
Cover of Sajó’s *Krieg und Frieden im Ameisenstaat.*

**Figure 5.**
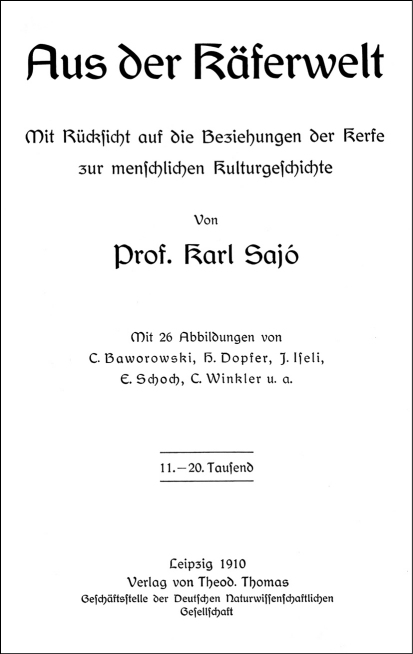
Front page of Sajó’s *Aus dem Leben der Käfer.*

Sadly, his writings have still to be explored in full. His communications appeared in 13 periodicals at home and 12 abroad, mainly in German. He was a senior staff member for 18 years, from 1895 to 1913, of *Prometheus*, the main German-language journal of popular natural history, published in Berlin, where 169 pieces, including some of his most important studies, appeared. Also published in Germany were three of his four books. He wrote the most successful about honey bees (*Unsere Honigbiene*; Sajó 1909, 1914, 1923a) and about ants (*Krieg und Frieden im Ameisenstaat*; Sajó 1905a, 1908, 1923b) (Figure 4); these appeared in almost 30 editions and 300,000 copies in Germany between 1905 and 1923. His ant monograph also appeared in Hungarian in a translation by Ede Früchtl (Sajó no date), and his bee work twice in Czech, through the cooperation of A. Muťovský and J. Kebrle (Sajó 1919). A major work of applied entomology by him that appeared in 1910 (Sajó 1910a) (Figure 5) also contained much cultural information connected with scarabaeoids and meloids, including a remarkably interesting idea that was cited in many later publications (Schimitschek 1968, Hogue 1983, Kevan 1985, Scholtz 2008): *“That the association between the dung ball of scarabaeids and wheels might be not so far fetched is documented by the vision of Ezekiel in the Bible (Ezekiel 1: 1–28). In his vision the prophet Ezekiel describes four cherub angels that resemble scarab beetles in several aspects including the metallic appearance, four wings with two different pairs, spines at the anterior limb pair, cleft feet, the back and forth movement, and the carrying of a wheel that is round in every direction. The idea that cherubim might represent scarab beetles originated from the Hungarian zoologist Sajó (1910) and was refined by the American cultural entomologist Hogue (1983). The interesting aspect of this hypothesis is that the so-called Ezekiel’s Wheel, which is often depicted as two wheels interlaced at right angles and carried by the cherubim, might be the transformed description of the dung pill. Thus, the association of a scarab dung ball with a wheel might not be a foreign thought of Middle Eastern ancient people”* (quoted from Scholtz 2008).

The first edition of his fourth book, *Blätter aus der Lebensgeschichte der Naturwesen* (Sajó 1911a) contained a collection of his papers. Subsequent editions are also known (Sajó 1911b, 1922). It was to be the first of a ten-volume collection of articles, but the other volumes never appeared, due to the First World War and the economic crisis that ensued.

Sajó kept up a very lively correspondence with specialists and institutions abroad. He was sending three or four letters or packets a day in the 1920s and early 1930s. These connections and writings of his did much to make the entomology of the sandy puszta of the Great Hungarian Plain known abroad (Sajó 1880a, 1882, 1883). He noted down observations with daily regularity. He himself stated that for many years, hardly a day went by without discovering something new or solving some old conundrum. It is a huge loss to universal entomology that he should have ceased his writing a quarter of a century before he died. For a long time, World War II Őrszentmiklós was a war zone, and his home and laboratory, his library of 3000 volumes, his unpublished notes, and all his correspondence were destroyed. All that remained of his library was a handful of volumes that his heirs presented to the Hungarian Museum of Natural Sciences, Budapest after his death.

The question arises: why should a man whose life had been imbued with research and research findings suddenly and irrevocably have laid down his pen? Two possible reasons were advanced by Szilády (1941). One was advancing years, the other a change of editor at *Prometheus*. Editor Otto Nicolaus Witt had great praise for Sajó’s writings in a letter of 18 April 1913: *“I had to read several thousand pages, but there was only one man whose writings it was always a joy to take up, for I knew that I could always find new knowledge and stimulation in them”* (cited in Szilády 1941). Sajó received even greater recognition from his readers, in the form of letters, congratulations and requests for advice. When Witt died and the new editor began to delay publishing his writings, Sajó became permanently disillusioned.

Sajó’s foresight was shown in 1894, when he was among the first to write about the role of insects in spreading disease (Sajó 1894a, 1894b, 1895a, 1898a, 1910b), predicting that *Anopheles*-mosquitoes would be found to spread malaria, and the African *Glossinia*-flies and the Kolumbács fly, *Simulium colombaschense* (Scopoli, 1780) several other diseases. This pioneering article appeared in *Prometheus*, whose editor drew readers’ attention in a footnote to the fact that it was the first article in the field to appear in Europe. It was cited in French papers also. Sajó wrote in the study that insects *“are not just the loving postmen of flowers,”* but *“heralds of the scythe of Death.”* His prediction became crystal-clear not long afterwards, as he noted that *“a very important and interesting field is opening up for bacteriologists.”*

Sajó dealt in his works with the connection between the weather and the behaviour of living organisms (Sajó 1896c, 1896d, 1896e, 1896f, 1897a, 1899). Although Sajó was not the first to recognize this, his name is associated with a deterministic approach to that connection, i. e. in the strong wind that precedes a storm, insects will rise up into the air and so produce a mass spread over longer distances, which is advantageous to the species (Sziráki 1985). For completeness’s sake it should be mentioned that the first detailed description of the phenomenon was made by South (1885), although his account was confined to the noctuid moth *Autographa gamma* (Linnaeus, 1758), whereas Sajó generalized it.

His report (Sajó 1896b), written in most enjoyable Hungarian prose, met with scarcely any reaction in Hungary, where contemporaries simply did not concern themselves with matters of a theoretical nature. For instance, Sajó’s discoveries about aestivation – covered later – were ignored for half a century. The next Hungarian entomologist to deal substantively with diapause was Gyula Sáringer, in conjunction with Tibor Jermy, from the 1950s onwards. On the other hand, the German versions of his papers were widely noticed in Europe and overseas. This is well exemplified in an article in the *American Naturalist* (Webster 1902): *“Relative to the concluding point in this paper*, viz., *the influence of wind and thunderstorms combined on insect diffusion, I beg to call attention to a most interesting series of papers contributed to* Prometheus, *a German scientific journal much like our* Scientific American, *by Prof. Karl Sajó, of Budapest, Hungary. Professor Sajó says that it is known that ‘*before thunderstorms the crayfish come out of the water into the grass on the banks of the river or lake; many fishes act as if they were insane, and many birds and mammalia become irritated and angry. Even the micro-organisms are subject to similar changes; for instance, before thunderstorms in late fall, the wine fermentation can reach so great a violence as to cause the fermenting juice to suddenly run out of the vats. The greater the change in the atmosphere, the greater the unrest of the living being …’ *Continuing, Professor Sajó calls particular attention to the ‘*great unrest and activity that takes place in the insect world just in the sultry hours preceding a thunderstorm, and to the fact that insects in the air at the time the storm bursts are driven like chaff to great distances, – perhaps into other countries, across rivers, lakes, and mountains; not only the species that fly but many that do not fly may thus be transported to new homes.’ *And again*, ‘Many Aphides creep to the crowns of the plants, then drop themselves at the proper moment into the violent current of the storm. A number of these insects land in places where there is no food supply for them and they die. A part of them reach places where their species is already established, and fare no better. Part are thrown into the water, sometimes in oceans, and perish. A proportionally small number arrive at such places as may be called really favorable for their diffusion, *viz.*, where the species has never established itself before, or, having done so, died out before the arrival of newcomers, and, therefore, natural enemies had not preceded them. Such individuals as are thus thrown into favorable places have a chance to multiply into large, populous colonies within a short space of time, and continue until their enemies find them out, or they become over-populous and devour all of their food supply, resulting in what to them is famine.’ *There is probably not an American entomologist who has not encountered illustrations similar to those enumerated by the writer of the above, and, while we may not have wholesale introductions of new things among us, there is no doubt that localities are often first colonized by certain kinds of insects in this manner, whereas the wind or the thunderstorm acting separately would not bring about such a condition of affairs. I have stated that, in applying trap lights or lanterns, or edible baits like sweetened sour beer, we, as a rule, secure males and spent females, but the influence of weather conditions that usually precede a thunderstorm (that is, a close, sultry condition) has the effect of bringing out both sexes, – a result due, so far as can now be determined, to some subtile action on their sexual life. As Professor Sajó so aptly illustrates this point, I will quote him again quite fully*: ‘What influence the weather has, especially on the activity sexual life, must be known to every zoologist; even man is not an exception from these “living barometers.” Not only children, not only the female sex, but the sick ones experience the influence of the weather on the functions, especially on the nervous system; and everybody without exception are thus influenced, though not all may be aware of the fact. The same causes that in many produce unrest and irritation render others dizzy, stupid, or sleepy, according to the temperament of the individual.’ *The effect of electricity on the nervous systems of insects, especially as relative to their love affairs, would constitute an interesting study, and one that ought to be carried out; but even as it is, we can see that the thunderstorm, in conjunction with the wind, may accomplish in the diffusion of insects that which neither element alone would bring about.”*

Sajó’s articles on the subject appeared mainly in German-language journals, and although most later references were made not to the original papers (for example Sajó 1897a) but to references in Uvarov’s *Insect and Climate* (Uvarov 1931), it is heartening to find several exceptions (Wellington 1946, Edwards 1961, Flitters 1963).

Burgeoning pathogens and insect pests caused increasing problems as agricultural production spread and intensified. How could these be contained without doing damage to the environment? Sajó recognized what a blessing the natural enemies of the pests represented to plant protection and was among the first to propose employing them (Sajó 1902g).

## Károly Sajó’s investigations into leaf-beetles

Sajó’s outlook on nature was decisive also in his researches into the life history of leaf-beetles. Although he broke ground with his discoveries about the life history of many harmful leaf-beetle species, his most important findings concerned the aestivation of *Entomoscelis adonidis* (Pallas, 1771), which he published in Hungarian (Sajó 1896g) and in German (Sajó 1896h, 1896i). He returned to the matter some years later, in the light of other findings (Sajó 1900a, 1900b, 1911c).

In 1895, Sajó began to investigate on his own estate at őrszentmiklós the life history of *Entomoscelis adonidis* (Pallas, 1771) and its larva, the so-called “black caterpillar”, which was a formidable pest of seed rape in Central and Southern Hungary in the 19th century. He recalled a letter that a farmer by name of Friedrich Rovara had sent to the Royal Hungarian State Entomological Station in the 1880s, stating that he had found developed examples of *Entomoscelis adonidis* (Pallas, 1771) in the soil. The staff had probably thought the communication was mistaken and taken no action. Not so Sajó, who placed specimens in an insectarium, where they did indeed disappear into the soil at the end of May and reappear only in October. Sajó established that this was a perfect case of aestivation, and managed to find the reason for it (Sajó 1896j). Although the phenomenon appeared at that time to be unique, Sajó was sure it must occur in some other species as well. This was soon confirmed: W. Kolbe (1899) augmented Sajó’s finding by discovering aestivation in two other leaf-beetle species: *Gonioctena viminalis* (Linnaeus, 1758) and *Chrysolina sanguinolenta* (Linnaeus, 1758). Sajó went on to experiment with other leaf-beetles, but without clearly confirming aestivation among them.

The discovery was a milestone as the first experimental observation of the phenomenon known today as aestivation. He also confirmed hibernation in a number of insects (Sajó 1896e, 1896f). The biological explanation for diapause he could not yet give, of course. He proposed a process of self-purification from metabolic intoxication in the insects (see also Bodenheimer 1952).

Sajó, as an out-and-out practical man, dealt mainly with the leaf-beetle pests in agriculture and horticulture. He presented the life history of *Oulema melanopus* (Linnaeus, 1758), and how to defend against it in several publications (Sajó 1890b, 1890c, 1893a, 1893b, 1894c). He was the first scientist in this country to investigate beetles inhabiting asparagus, notably the *Crioceris* spp. (Sajó 1895b, 1897b, 1902d, 1902f).

He also presented in shorter papers the life history of *Xanthogaleruca luteola* (O. F. Müller, 1766) and *Gonioctena fornicata* (Brüggemann, 1873), in Sajó 1888 and 1892 respectively, along with the damage they did and how to protect against it.

While working on protection against phylloxera, Sajó lit on damage done by the Western Grape Root-Worm, *Bromius obscurus obscurus* (Linnaeus, 1758). He was occupied for some time with its life history and range of food plants (Sajó 1891b, 1896k, 1898b), and with the taxonomic problems it raised. When dealing with the latter, he noticed that of the two morphologically differentiable taxa (*“Eumolpus obscurus”* and *“Eumolpus vitis”*), *“Eumolpus obscurus”* never occurred on grape vines, even though the investigations had extended to wine regions throughout the Carpathian Basin. He collected *“Eumolpus obscurus”* solely from *Epilobum* species, mainly in hilly and mountainous habitats and beside streams. He emphasized repeatedly that *“Eumolpus obscurus”* and *“Eumolpus vitis”* are two separate species, although they were being treated as one at that time. He stressed that life history and length of development needed to be considered when distinguishing the species. He raised the question again in a lecture delivered to the Zoological Department of the Royal Hungarian Natural History Society on 13 April 1893 (Sajó 1893c). He drew a parallel with the species pair *Entomoscelis adonidis* (Pallas, 1771) and *Entomoscelis sacra* (Linnaeus, 1758), because he did not think it was justified to amalgamate two species simply because there existed transitional forms between them. He showed that the habitats, food plants and time of appearance of the two species differed markedly.

In 1896, an analysis of the *Eumolpus* species by Émile E. A. Topsent appeared in the *Bulletin de la Société d’ Etüde des Sciences naturelles de Reims*. Sajó quickly put up an opposing view (Sajó 1897c): Topsent’s hypothesis, that *“Eumolpus vitis”* and *“Eumolpus obscurus”* are one and the same, and that the difference in colour is due to the food plant from which each may chance to be taken, was shown to be without the support of facts or even an investigation.

Surprisingly, this problem has still not been solved. According to many authors (Kaszab 1962, Mohr 1966, Gruev 1992, Gruev and Tomov 1984, 1998, Jelínek 1993, Vig 2002) the *villosulus* (Schrank 1781) variation (= *vitis* auct. nec. Fabricius 1775) is a separate subspecies, and the taxonomic structure is *Bromius obscurus obscurus* (Linnaeus, 1758) and *Bromius obscurus villosulus* (Schrank, 1781). More recently the view has spread that the distinction is unjustified (Warchałowski 2003, Moseyko and Sprecher-Uebersax 2010), although I am not familiar with the actual evidence for saying so.

## Károly Sajó’s work on taxonomy

Károly Sajó had wide interests, but his prime focus was on applied entomology; his work in classic taxonomy is modest. His main concerns were the Hymenoptera and Hemiptera, and among the beetles of Coccinellidae.

In the Őrszentmiklós district, he collected four specimens of a sphecid wasp that he described as a new species named *Oxybelus treforti* (Sajó, 1884). (This is now classified as the subspecies *Oxybelus argentatus treforti* Sajó, 1884; see Bohart and Menke 1976). Although he was the first to collect a specimen of *Oxybelus aurantiacus*, he conceded the right of describing it to Sándor Mocsáry (Mocsáry 1883). He was also the first to discover the macropterous forms of the Hemiptera *Blissus doridae* Ferrari, 1874 and a *Plinthisus hungaricus* Horváth, 1875 (now a synonym of *Plinthisus longicollis* Fieber, 1861) (1880b, 1880c, 1880d, 1880e).

In line with his period, that paid great attention to describe aberrations and variations (Weise 1882), he also described numerous ladybird variations (Sajó 1880f, 1881), which count now as synonyms or invalid categories (Leman 1922, Kovář 2007).

Sajó possessed a huge collection of insects, from which he sent significant amounts abroad, in an attempt to relieve his financial problems after the First World War and during the Great Depression (personal communication of Alfred C. Kinsey, cited by Balás and Sáringer 1982). There is confirmation of this in the Scientific Notes and News column of the 12 June 1925 number of *Science* (Kinsey 1925): *“The death of Dr. Karl Sajó of Hungary was reported during the year of 1924. I have a characteristic letter from Professor Sajó, dated April 23, and I am delighted to be able to make this correction. Dr. Sajó has been rendering a good service to American entomologists by offering Hungarian insects for sale, and I hope that this mistaken report has not interfered with his work.”*

Not long ago, about a thousand beetle specimens originating from Hungary were found in the collection of the Sam Noble Oklahoma Museum of Natural History, part of the University of Oklahoma (email letter from Katrina Menard, collections manager of recent invertebrates). Based on the collecting labels, some of these were collected in 1909–11 in the district of Őrszentmiklós, and many specimens also bear the name Károly Sajó. There can be no doubt that they formed one of the consignments he sent to the United States.

Shortly after Sajó’s death, his insect collection was presented by his family to the Hungarian Natural History Museum in Budapest, where it can still be studied. One item from it was a specimen of *Adapsilia coarcata* Waga, 1842 (Pyrgotidae), which added a new species and a new family of flies to the fauna of Hungary (Soós 1943). Indeed Sajó collected between May and September the only specimens of the family yet found in Europe. Árpád Soós, a celebrated dipterist, found specimens from Sajó, collected at Őrszentmiklós, in the Thalhammer Collection in Kalocsa. There also the labelling shows that János Thalhammer received these from Sajó.

Sajó was concerned with problems of classifying insects and the proliferation of synonyms (Sajó 1900c). He criticized the habit of awarding different specific names within a genus based only on differences of form. He also disapproved of those who published specific descriptions in little-known, small-circulation periodicals, which he believed was partly responsible for this proliferation of synonyms.

## Károly Sajó’s nature protection work and the beginnings of nature protection in Hungary

The earliest initiatives in Hungary for protection of the environment date back to the early 19th century, although these relied on the personal activity of a few individuals and did not take any institutionalized form. In 1841, János Salamon Petényi, an eminent zoologist, was almost the first to warn that the buffalo of the Carpathian Basin were dying out and so were the beavers (Petényi 1847)^1^. At a meeting of the Royal Hungarian Natural History Society in 1866, János Kreisch called for protection of the Tatra marmot, *Marmota marmota latirostris* Kratochvil, 1961. Such isolated initiatives had little success, for they lacked a scientific backing. More effective legislative assistance in protecting the environment came with the game laws (IV/1872 and XX/1883). These defined close seasons and listed the species that could be hunted. Those seen as beneficial (from the hunting and other economic points of view) were given protection and those seen as detrimental could be shot “at any time”, but debates around the concepts of benefit and detriment caused further uncertainty. Apart from the game laws, there appeared in 1888 a ministry order (32.042/1888. FIK) giving protection on crown land to Pallas’s sand grouse, *Syrrhaptes paradoxus* (Pallas, 1773), a species found normally on the Asian steppes, and seeking to encourage its presence. This was done purely on grounds of natural value, regardless of benefit or detriment, which was to be gauged by subsequent research. This makes the 1888 order the first action by the state to have an express purpose of nature protection.

The social and economic efforts towards a nature protection movement in Hungary had succes at the turn of the 19th and 20th centuries thanks to the presence of a social demand and basis for it. This receptive milieu gave support to a concept of nature protection that had been proposed on a specialist level, with Károly Sajó playing a formulating part in it. Even in his early writings, he was listing animal and plant species that were in danger of extinction. In 1905 he was outlining the main steps to require to “rescue the treasures of virgin nature” (Sajó 1905b). Here two underlying ideas appear: that all living organisms should be preserved in their original environment, and that the cause of nature protection had to be furthered through legislation. He made a specific proposal: that there should be purchased out of the annual state budget “national protected areas” that are “interesting and excellent for their fauna or their flora or their geological features, or even their natural beauty.” He also described in detail how protected areas in various parts of the country might be chosen from state-owned estates, and their preservation be placed in the hands of nature protection guards drawn mainly from the forestries. He issued an appeal to Hungarian society in the meantime, before the “national protected areas” were designated, for scientists and others with a feeling of responsibility for nature at least to refrain from shooting rarer mammals and birds, and made bold and frank criticisms of them, primarily for the responsibility they bore for the irreversible damage and destruction wrought by procrastinating over the protection of the country’s natural assets: *“Let it not be science, not men of science who destroy the treasures of nature, but only those who still have no notion of what marvellous, multifarious masterpieces cover the surface of our globe: masterpieces whose effects are irretrievable, for the crude works of man cannot even approach the delicacies of the organic and inorganic realm. For we scientists of the last century have indeed been at fault! At fault in not striving with enough energy the veto of science whenever we saw a wave of total destruction overwhelm in a few years works that had taken Mother Nature millions of years to produce, and allowing destruction to plumb the depths”* (Sajó 1905b).
However, Petényi’s appeal for the European buffalo, Bison bonasus (Linnaeus, 1758) came too late: the last specimen in Hungary was killed in 1762 on the Plaj heights in Borgó, although a note at the time puts the extermination of the last specimen later, in 1814, on the Udvarhely side the Madaras Hargita. The beaver fared no better: the last specimen was shot a few years later near Ács, in 1854.

Sajó considered that the species of plains and hills and watery habitats were in the greatest danger. He devoted a separate article to the exceptional importance of primary forests (Sajó 1905c). He intended an important role in the operation of nature protection to be played by professional societies, especially in the natural sciences. He assigned to them above all the role of enlightenment and propaganda, in the hope that their actions would also encourage various private initiatives (Lukács 1976).

Sajó settled near Őrszentmiklós in the period after the phylloxera epidemic. He was appalled to notice the extent to which viticulture on sandy soil had been at the expense of the original plant cover and the beetle community associated with it (Sajó 1893d), and so he established a reservation on five cadastral *hold* (3 hectares) of his estate. This to the author’s knowledge was the first territory in Hungary to be set aside for purposes of nature protection, on which Sajó gave ecological stability priority over farming. In that respect he was the first active Hungarian practitioner of nature protection. He was the first to ask how the damage from pests and diseases could be reduced without damaging the environment. Following the example of the famous *Pozsony Garden* of the Jesuit János Lippay (Lippay 1664), he established several noble exotic and indigenous tree and shrub species on the alkaline dunes (Sajó 1896l, 1902h). The marked out wider than usual roads on his estate and would not allow the verges to be mown, so as to protect the plant and insect life living there. Unfortunately, this nature protection area, unique in Hungary and even in Europe, was neglected after this death.

However, it was not so much by his personal example as through his writings that he furthered the cause of nature protection at home and elsewhere in Europe. He spoke out against the damage caused by habitual hunting, inordinate collecting, and unplanned afforestation. His articles spoke of the national parks that already existed in North America and Africa and of the initial efforts at nature protection by European nations.

Károly Sajó’s writings also contributed to the conceptual foundations for nature protection and for political decision-making in Germany (Genath 2005). Based on Sajó’s contributions to *Prometheus* and on personal experiences of natural destruction, Wilhelm Wetekamp argued in the Prussian provincial assembly in 1898 that a network of national parks based on those in North America should be set up (Piechocki 1998). Wetekamp’s speech prompted Agriculture Minister Hugo Conwentz to commission him to survey the Prussian forests, and his report (Conwentz 1900) can be considered the first nature protection inventory (Sajó 1900d). The initial steps were being taken in Europe as well …

In 1905, the year in which Sajó’s famous study of the “treasures of all nature” appeared, the subject of nature protection was regularly discussed at meetings of the Zoological and Botanical Department of the Royal Hungarian Natural History Society. There were several debates within the society, culminating in a request to the board that it should seek legislature on the protection of natural assets. The resulting proposal was backed also by the Hungarian Geographical Society and the National Forestry Association. Károly Kaán proposed to the Royal Hungarian Natural History Society in 1907 that an appeal be made to the agriculture minister on the subject of protecting “the primary forests, the spectacular museums of nature”. Kaán’s proposal provided the basis for a detail plan of action from the National Forestry Association and the Royal Hungarian Natural History Society, which emphasized measures to protect the oak woods of the plains and hills and the gallery forests along the Danube. Thereafter, the work of Agriculture Minister Ignác Darányi and of Károly Kaán led to the passage of the first nature protection legislation in 1909.

## Epilogue

Károly Sajó inhabited and influenced at the turn of the 19th to the 20th century a world that was undergoing radical transformation. He was a pioneer in the true sense, whose many discoveries and innovative proposals, and whose whole outlook opened up new fields in applied entomology. Sadly, he never received the recognition he deserved in his own country, though he remained an out-and-out Hungarian entomologist, who gained great prestige for his country. In many cases his contemporaries failed to grasp his outstanding achievements and pioneering discernments, and recognition of their importance had to wait until the 1950s. Even today, his writings have not been fully charted, but most of those on the subject of plant protection are referenced fully in Balás and Sáringer (1984) and in the appropriate chapters of the six-volume manualedited by Tibor Jermy and Klára Balázs (1988–1996). His wide interests, his writings, his intellectual heritage and his outlook on life may well justify the conclusion reached by Nagy (1992): Károly Sajó was Hungary’s Jean-Henri Fabre.
